# Optical Properties and Conductivity of PVA–H_3_PO_4_ (Polyvinyl Alcohol–Phosphoric Acid) Film Blend Irradiated by γ-Rays

**DOI:** 10.3390/polym13071065

**Published:** 2021-03-28

**Authors:** Susilawati Susilawati, Saiful Prayogi, Muhamad F. Arif, Noor Maizura Ismail, Muhammad Roil Bilad, Muhammad Asy’ari

**Affiliations:** 1 Master of Science Education Program, University of Mataram, Jl. Majapahit No. 62, Mataram 83125, Indonesia; 2 Physics Education, FKIP, University of Mataram, Jl. Majapahit No. 62, Mataram 83125, Indonesia; 3 Faculty of Applied Science and Enginering, Universitas Pendidikan Mandalika UNDIKMA, Jl. Pemuda No. 59A, Mataram 83126, Indonesia; saifulprayogi@ikipmataram.ac.id (S.P.); muhammadroilbilad@ikipmataram.ac.id (M.R.B.); muhammadasyari@ikipmataram.ac.id (M.A.); 4 Department of Materials Engineering, Institut Teknologi Sumatera, Lampung Selatan 35365, Indonesia; mf.arif@mt.itera.ac.id; 5 Faculty of Engineering, Universiti Malaysia Sabah, Jln UMS, Kota Kinabalu 88400, Malaysia

**Keywords:** optical properties, conductivity, gamma irradiation, polymer film blend

## Abstract

This study assesses the optical properties and conductivity of PVA–H_3_PO_4_ (polyvinyl alcohol–phosphoric acid) polymer film blend irradiated by gamma (γ) rays. The PVA–H_3_PO_4_ polymer film blend was prepared by the solvent-casting method at H_3_PO_4_ concentrations of 75 v% and 85 v%, and then irradiated up to 25 kGy using γ-rays from the Cobalt-60 isotope source. The optical absorption spectrum was measured using an ultraviolet–visible spectrophotometer over a wavelength range of 200 to 700 nm. It was found that the absorption peaks are in three regions, namely two peaks in the ultraviolet region (310 and 350 nm) and one peak in the visible region (550 nm). The presence of an absorption peak after being exposed to *hυ* energy indicates a transition of electrons from HOMO to LUMO within the polymer chain. The study of optical absorption shows that the energy band gap (energy gap) depends on the radiation dose and the concentration of H_3_PO_4_ in the polymer film blend. The optical absorption, absorption edge, and energy gap decrease with increasing H_3_PO_4_ concentration and radiation dose. The interaction between PVA and H_3_PO_4_ blend led to an increase in the conductivity of the resulting polymer blend film.

## 1. Introduction

Polymer blending with another material can be used as an effective method to alter the resulting film blend properties. An insulating polymer such as poly(vinyl-alcohol) (PVA) can be turned conductive by blending with other materials to diversify its applications. Previous studies have shown that polymer-based synthetic materials can be applied into electrical and optical devices [1], such as photovoltaic devices [2], rechargeable batteries and nonlinear optical devices [3], and light-emitting diodes (LEDs) [4]. Moreover, conductive polymers have also been applied in the health sector, including as a biosensor [5] and a coating agent for detecting cancer cells [6].

Polymer materials generally have a high degree of flexibility for various end-products [7]. In this study, we used PVA as the host polymer because it is water-soluble [8]; has good mechanical properties [9,10]; is non-toxic and elastic [11]. PVA is an insulator because it does not have a charge functional group such as sulfonic acid (–SO_3_H) or carboxylic (–COOH) groups [12]. Therefore, an acid group donor has the potential as a dopant of PVA mixture to increase its conductivity [13], one of which is phosphoric acid (H_3_PO_4_). Polymer film PVA–H_3_PO_4_ complexes have been investigated and it was found that the amorphous structure of PVA increases with increasing concentration of H_3_PO_4_ with the H+ cation mobility of $\mu_{H^{+}}≅$ 1.3 × 10^−4^ cm^2^ V^−1^ s^−1^ [14].

The potential of PVA and phosphoric acid polymeric film blends in many applications has long been explored [14,15], with emphasis on optical properties [16], electrical conductivity [17,18,19], and/or both [20]. The specific application of polymers from a mixture of PVA with phosphoric acid is mainly as an electrochromic device [20] and a component in polymer batteries [18]. Polymer films of PVA material mixed with acidic compounds and irradiated with electron beams were also investigated for its application as radiation dosimetry [21]. For this reason, this polymer material is intensively researched. Previous studies have shown that the favorable conductive property of PVA film was found when it is mixed with phosphoric acid [19]. The conductivity of the polymer film increases with an increasing concentration of phosphoric acid in the blend [22]. The resulting polymer films were also found to have good thermal and mechanical properties [17,23]. However, the blending of PVA with phosphoric acid accompanied by irradiation—to our best knowledge—has not been reported in the literature, and thus addressed in this study.

Currently, polymer material processing and modification technology explore the use of radiation techniques, one of which is γ-ray irradiation [21], because it is considered more practical when compared to the traditional chemical processes. The radiation process can affect polymerization, cross linking, grafting and chain breaking [24]. Therefore, the characteristics of polymer materials due to γ-ray irradiation need to be explored further. This study explores the optical properties and conductivity of PVA–H_3_PO_4_ (polyvinyl alcohol–phosphoric acid) polymer film blend irradiated by gamma (γ) rays so that the blend can be used in diverse applications.

## 2. Materials and Methods

Before optical and electrical conductivity measurement, the polymer film blend was prepared from PVA (M_w_ of 72,000 g/mol, Merck, NJ, USA), and phosphoric acid solution (H_3_PO_4_) (M_w_ of 98 g/mol, Merck, NJ, USA) at 75 v% and 85 v%. The polymer film blend of PVA–H_3_PO_4_ was prepared via the solvent-casting method by dissolving 35.5 g of PVA into 700 mL of deionized water, then heated at 90 °C and stirred using a magnetic stirrer (AS-ONE, Japan) for 4 h until the PVA solution was formed, followed by natural cooling. The 75 and 85 v% of H_3_PO_4_ solutions were then prepared through dilution. A 100 mL PVA solution was mixed with 10 mL of 75 v% and 85 v% of H_3_PO_4_ solution, then stirred for 2 h. After a homogeneous solution was formed, the solution was transferred to a glass box for drying. The drying was carried out for 1 month to form a PVA–H_3_PO_4_ polymer film blend. The polymer film blend of PVA–H_3_PO_4_ was subsequently cut into a few specimens of 4 cm × 4 cm and was irradiated with gamma ray (γ) from a source of Cobalt-60 isotope using Irradiated Natural Rubber Latex (Rubberfoam, Indonesia) of 1.25 MeV. The average thickness of each film was about 0.22 × 10^−3^ m. The sample of polymer film blend was irradiated under different dosages of 0 kGy, 5 kGy, 10 kGy, 15 kGy, and 20 kGy.

The optical property of the polymer film blend was measured using a Spectrophotometer (LW-UV- 200 RS Spectrophotometer, single beam, mrclab, Israel) with a minimum wavelength of 200 nm, maximum wavelength of 800 nm, and power of 110/220 VAC, 50/60 Hz. Optical absorption characteristics were plotted in the form of a graph to show relationships between wavelength and optical absorption. The absorption edges were evaluated from the absorption coefficient (α(υ)) according to the Urbach edges method [25,26], and the optical energy gap (Eg) was determined according to the Mott and Davis’s model [27].

The electrical conductivity of the polymer film blend was measured using a resistivity meter (surface resistivity meter) according to ASTM D 257-99 (potential electrode 0.5 kV). Before analysis, the sample was conditioned in a chamber at a temperature of 23 °C and relative humidity of 50% for 40 h. The measurement time was less than 1 min at a temperature of 23.5 °C and relative humidity of 58.0%.

## 3. Results and Discussion

### 3.1. Optical Absorption

**Figure 1:**
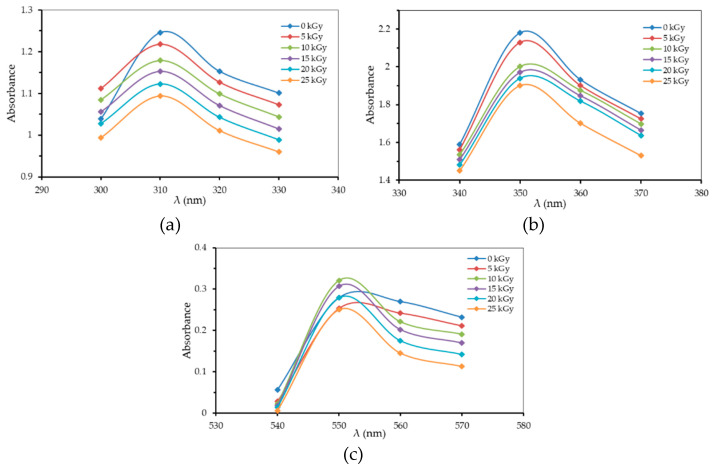
The relationship between absorbance (A) and wavelength (λ) for phosphoric acid (H3PO4) 75 v% concentration for each radiation dose: (**a**) λ = 310 nm (peak I); (**b**) λ = 350 nm (peak II); (**c**) λ = 550 nm (peak III).

The results of measuring samples from the PVA–H_3_PO_4_ polymer film blend irradiated with γ-rays at different radiation doses with two concentrations of H_3_PO_4_ (75 v% and 85 v%) obtained the maximum absorption values at wavelengths of 310 nm (peak I), 350 nm (peak II), and 550 nm (peak III) for all radiation doses and concentrations. The relations between optical absorption (absorbance) (A) and wavelength (λ) for the H_3_PO_4_ 75 v% concentration for each radiation dose are presented in Figure 1.

Figure 1 shows that the 75 v% PVA–H_3_PO_4_ polymer film material has a wide absorption area in the range of 300–330 nm (with maximum peak at 310 nm), 340–370 nm (maximum peak at 350 nm), and 540–570 nm (maximum peak at 550 nm). This was consistent with both non-radiated (0 kGy) and gamma irradiated films of up to 25 kGy. The absorption peaks for pure PVA films were found at wavelengths of 204, 277, and 324 nm. When the PVA films were phosphorylated, the characteristics of the films were configured in three wavelength ranges, isotactic (389–398 nm), syndiotactic (418–420 nm), and atactic (440–446 nm), as reported elsewhere [16]. Previous studies showed the optical absorption peak of PVA–H_3_PO_4_–methylene blue film was observed at 425 nm [28]. The characteristics of the PVA film polymer absorption differed from one another depending on the mixing material, such as the polymer film of PVA–trichloroacetic acid–methylene blue (PVA–TCA–MB) resulting in three maximum absorption peaks at wavelengths of 360, 440, 560 nm [21]. The maximum absorption peak of the PVA–ZnO nanocomposite film was found at 506 nm [29], PVA–carbol fuchsin–crystal violet (PVA–CF–CV) polymer film at 560 nm [30], while PVA–CaF_2_ nanocomposite films at 300 nm [31].

**Figure 2:**
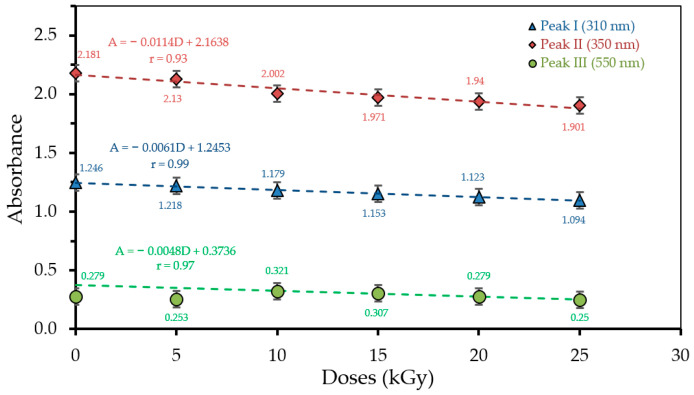
The relationship between radiation dose and absorbance of each λ (310, 350, and 550 nm) for H_3_PO_4_ 75 v% concentration. For peak III, the first two absorbance data were considered as outliers when plotting the trendline.

The relationships between radiation dose and absorbance of each wavelength (310, 350, and 550 nm) for H_3_PO_4_ 75 v% concentration are shown in Figure 2. The optical absorption values for all absorption peaks decrease with increasing radiation dose. The radiation dose relationship with optical absorption for the first peak is A = −0.0061D + 1.2453 (r = 0.99); second peak A = −0.0114D + 2.1638 (r = 0.93); and third peak A = −0.0048D + 0.3736 (r = 0.97). It can be seen that at a radiation dose of 0 kGy, the second absorption peak has the highest absorption value of 2.181 (a.u) followed by the first and third absorption peaks of 1.245 and 0.279 a.u, respectively. The absorption value decreases with increasing radiation dose. At a radiation dose of 25 kGy, the decrease in optical absorption at the first, second, and third peaks were 1.094, 1.901, and 0.250 a.u, respectively. These results are consistent with the earlier findings, in which the optical absorption value decreased with increasing doses of gamma radiation on the PVA–TCA–MB polymer film under the dose range of 0–14 kGy [21]. Likewise, the optical absorption value of the PVA–CF–CV polymer film at a wavelength of 560 nm was found to decrease with increasing dose of gamma radiation in the range 0–70 kGy [30]. Pure PVA films were also found to decrease with an increasing dose of gamma radiation in the 0–15 kGy range [32].

**Figure 3:**
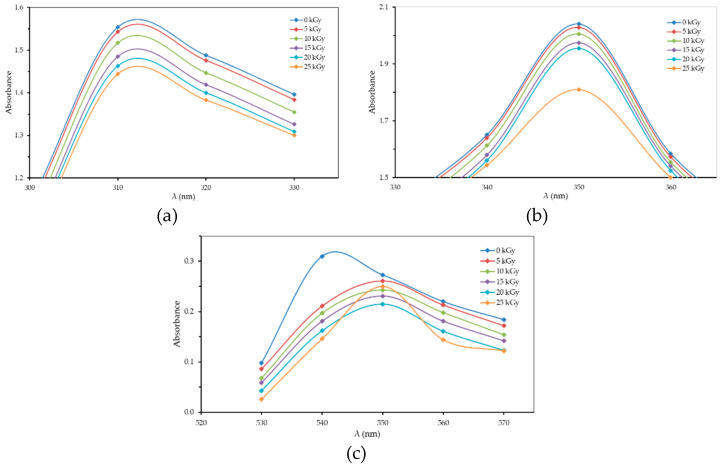
The relationship between absorbance (A) and wavelength (λ) for H_3_PO_4_ 85 v% concentration for each radiation dose: (**a**) λ = 310 nm (peak I); (**b**) λ = 350 nm (peak II); (**c**) λ = 550 nm (peak III).

In this present study, the optical absorption characteristics of the gamma irradiated PVA–H_3_PO_4_ polymer film were evaluated by varying the H_3_PO_4_ concentration. The relations between absorbance and wavelength (λ) for the H_3_PO_4_ 85 v% concentration for each radiation dose are presented in Figure 3. The optical absorption peak for the polymer film from the PVA–H_3_PO_4_ 85 v% concentration did not change when compared to the H_3_PO_4_ 75 v% concentration, where the optical absorption peak remained at 310, 350, and 550 nm. The shift in peak location may have occurred but was unnoticed, indicating of no major change in chemical bond (i.e., breaking bond). Similar finding was also found in the polymer composite PVA–poly acrylic acid–Al_2_O_3_, where variation in the concentration of Al_2_O_3_ did not alter the absorption peak at 300 nm [33]. The optical absorption peak was found to be consistent under blending of 23% and 57% chloral hydrate in the PVA polymer film [24], and with nickel variation in the PVA–polyaniline–Ni polymer film [34]. Similar to the present study, previous reports observed that the intensities of absorption were changed due to changes in the concentration of additive into the PVA film and the dosage of gamma ray radiation exposure [24,33,34,35].

**Figure 4:**
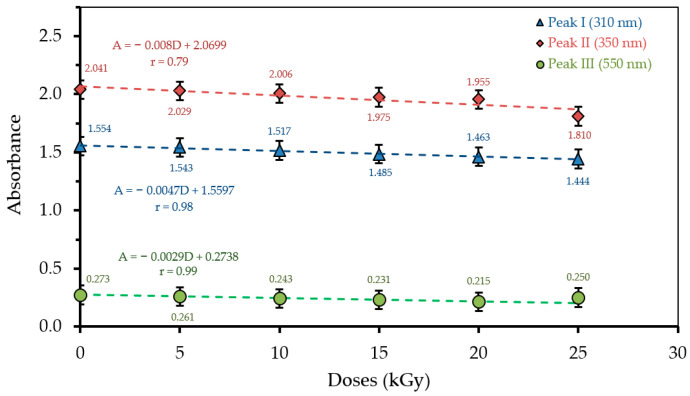
The relationship between radiation dose and absorbance of each λ (310, 350, and 550 nm) for H_3_PO_4_ 85 v% concentration. For peak III, the last absorbance datum was considered as outliers when plotting the trendline.

The relationship between radiation dose and absorbance for each absorption peak (310, 350, and 550 nm) for H_3_PO_4_ 75 v% concentration is presented in Figure 4. It shows that for the H_3_PO_4_ 85 v% concentration, the optical absorption values for all absorption peaks decrease with an increasing radiation dose with the relationship for each peak as follows: the first peak A = −0.0047D + 1.5597 (r = 0.98); second peak A = −0.008D + 2.0699 (r = 0.79); and third peak A = −0.0029D + 0.2738 (r = 0.99). The trend on the optical absorption for each peak for dosages of 0–25 kGy are 1.554 to 1.444 a.u for the first peak, 2.041 to 1.810 a.u for the second peak and 0.273 to 0.250 a.u for the third peak.

The trend on the effect of irradiation dosage on the absorbance peak can be explained as follows. The absorption process occurs when photons (with an energy greater than the energy of band gap) are absorbed by the material. It results in electron–hole pairs (excitons) [36]. The absorption peaks in the UV and visible regions are mainly caused by a transition of electrons from the valence band to the conduction band in polymer materials [35]. A similar phenomenon was expected of the polymer film PVA–H_3_PO_4_ developed in the present study after being exposed by irradiation energy (*hv*) in the form of electromagnetic waves at a wavelength of 310 nm (first peak), 350 nm (second peak), and 550 nm (third peak).

In this experiment, the optical absorption value for pure PVA was not measured, but previous studies showed that the optical absorption peak for pure PVA was also in the UV region [37,38]. The blending of H_3_PO_4_ into PVA was expected to form of the complex PVA–H_3_PO_4_, which can be evaluated by IR, NMR and XRD spectra [39]. The presence of an optical absorption peak in the visible light region at a wavelength of 550 nm can be attributed to the doping of H_3_PO_4_ into the polymer sample, which allows for the transfer of complex charges on the polymer film sample due to exposure of irradiation energy (*hν*) exposure.

The results found in this data set are in line with several previous studies. Devi et al. [40] developed a polymer film from a mixture of PVA with AgNO_3_ and studied optical absorption using a UV–VIS–NIR spectrophotometer. The results show that the optical absorption peak for pure PVA was in the UV region with a wavelength of 273 nm, but the presence of 25% AgNO_3_ doping in the polymer sample caused a new peak in the optical absorption measurement at a wavelength of 413 nm (visible light region), which indicated the presence of transfer of charge in polymer samples. The presence of multiple peaks in the measurement of the optical absorption value also indicates the excitation and recombination of the free charge carriers.

In general, the absorption peaks are in the UV and visible region, where in this study the optical absorption value decreases with increasing radiation dose. This trend suggests the occurrence of excitation and causes electron transitions between molecules in polymer bonds, namely the transition of electrons from HOMO to LUMO [41]. HOMO (highest occupied molecular orbital) is the top part of the state occupied by electrons in the valence band, and LUMO (lowest unoccupied molecular orbital) is the lowest part of the state that is not occupied by electrons in the band, or it can be said that HOMO is an analogue to the valence band, whereas LUMO is an analogue of the conduction band [34].

From the optical absorption data, it can be stated that the radiation dose and the concentration of the polymer film greatly affect the optical absorption value, where it decreases with increasing radiation doses and H_3_PO_4_ concentrations. Some reports claimed that γ-ray irradiation plays an important role in the pattern of breaking bonds in the polymer film which causes free charge, free ions, free radicals, and electrons [42,43], where the released electrons undergo a transition as a result of energy exposure (*hv*) at certain wavelengths [44]. However, in the present study, the radiation dose and H_3_PO_4_ concentration most likely intensify the interaction between PVA and H_3_PO_4_ due to their phase separation and dehydration.

### 3.2. Absorption Edge

**Figure 5:**
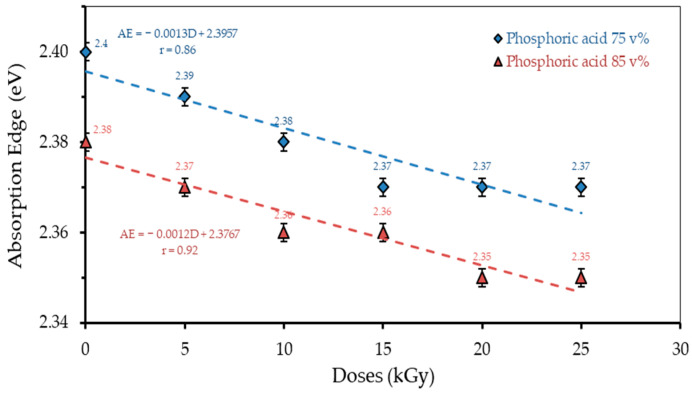
The relationship of the absorption edge with different radiation doses and H_3_PO_4_ concentrations.

The relationship between the radiation dose and absorption edge for each concentration of H_3_PO_4_ are presented in Figure 5. The absorption edge of polymer film with a H_3_PO_4_ concentration of 75 v% of 2.4 eV (dose 0 kGy) decreases to 2.37 eV at a radiation dose of 25 kGy. Likewise, for the H_3_PO_4_ 85 v%, the absorption edge decreases with an increasing radiation dose. The absorption edge shows the maximum energy absorbed by the polymer film PVA–H_3_PO_4_. It was found that the absorption edge value decreased with an increasing radiation dose and H_3_PO_4_ content in the polymer film. These results are relevant to previous studies, where the absorption edge decreased with an increasing radiation dose and CH composition in the PVA–CH polymer film [15]. The irradiated ion charge has a significant effect on the absorption edge shift in the UV–Vis spectrum as a result of the expanded conjugated system growth through the formation of radiation-induced intermolecular helical structures [44]. Several previous studies have shown that the absorption edge of the UV–Vis transmission spectrum in polymers shifts with an increasing influence of radiations [45,46,47,48].

### 3.3. Energy Gap

**Figure 6:**
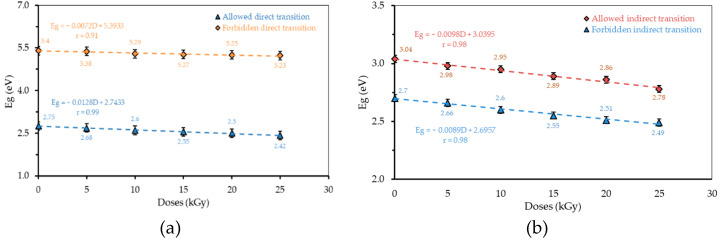
The relationship between the radiation dose and *Eg* for each transition of H_3_PO_4_ 75 v%: (**a**) direct transition; and (**b**) indirect transition.

**Figure 7:**
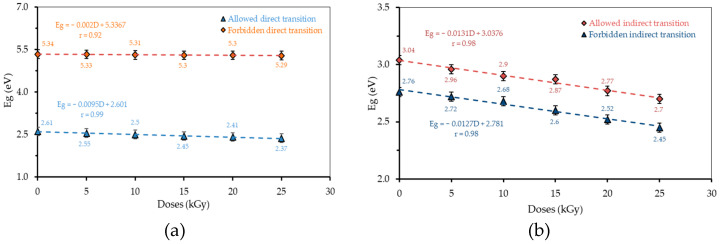
The relationship between the radiation dose and *Eg* for each transition of H_3_PO_4_ 85 v%: (**a**) direct transition; and (**b**) indirect transition.

The energy gap (*Eg*) is the energy band gap between the valence band and conduction band. The energy gap consists of four types of electron transitions, namely; the allowed direct transition (αhυ)^1/2^; the allowed indirect transition (αhυ)^2^; the forbidden direct transition (αhυ)^3/2^; and the forbidden indirect transition (αhυ)^1/3^ [24]. The relationships between radiation dose and *Eg* for each transition of H_3_PO_4_ 75 v% and 85 v% are presented in Figure 6 and Figure 7, respectively.

**Figure 8:**
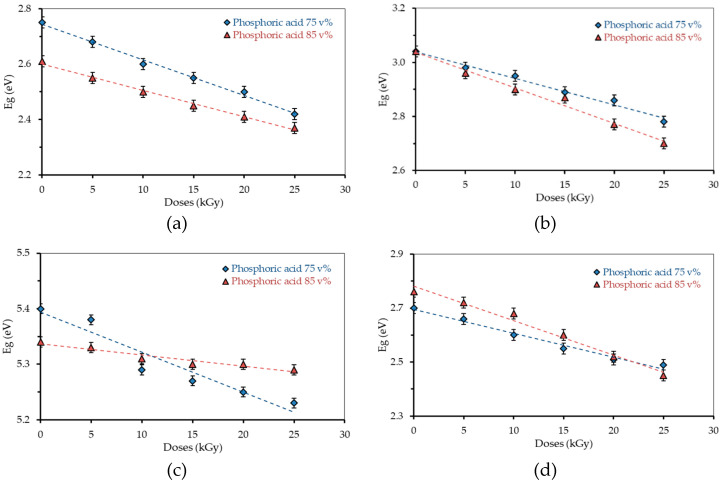
Energy gap at various concentrations and radiation doses: (**a**) the allowed direct transition (αhυ)^1/2^; (**b**) the allowed indirect transition (αhυ)^2^; (**c**) the forbidden direct transition (αhυ)^3/2^; and (**d**) the forbidden indirect transition (αhυ)^1/3^.

At each transition, a gap energy is obtained which varies according to the radiation dose. The energy gap value decreases with the increasing dose of γ-ray irradiation. The differences in the value of the energy gap for the variation in concentration and radiation dose for each transition are presented in Figure 8.

The results show that a higher H_3_PO_4_ concentration in the polymer sample lowers the energy gap. It implies that the addition of H_3_PO_4_ can alter the conductivity property of the polymer film to be conductive or non-conductive [34]. As detailed earlier, radiation coupled with higher H_3_PO_4_ concentration leads to a more intense interaction between PVA and H_3_PO_4_. The radiation has an important role in altering the chemical bonds of a polymer film, leading to formation of free ions, free-radicals, and free electrons, as reported elsewhere [48], in which the electron can release spectra of emission when transferred from the donor to the acceptor, which lowers the energy gap [24,49]. In the present study, no chemical bond breaking is expected as a result of radiation, judging from the absence of a peak shift from the UV–VIS absorption. Previous research revealed that the addition of metal, semiconductor particles and complex transition metal can manipulate the optical properties of polymer film [26,50,51,52].

### 3.4. Electrical Conductivity

**Figure 9:**
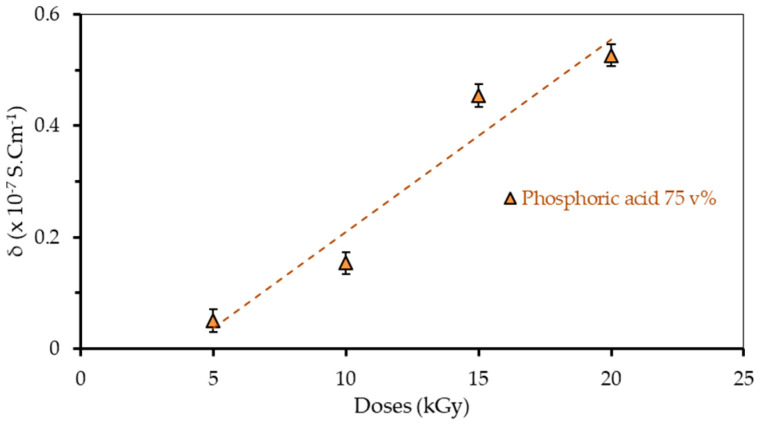
Electrical conductivity of the polymer film blended with H_3_PO_4_ 75 v%.

Electrical conductivity (σ) indicates the ability of a material to conduct electricity. Electrical conductivity can be determined by looking at the resistivity of a material where σ = 1/ρ. The electrical conductivity of the polymer film was in accordance with the results of the electrical resistivity (ρ) test of the polymer film for different doses (5, 10, 15, and 20 kGy) and the H_3_PO_4_ 75 v% concentration, as presented in Table 1 and Figure 9.

Figure 9 shows that the conductivity of the PVA–H_3_PO_4_ film increases with increasing radiation dosages. The concentration of H_3_PO_4_ also plays a role in determining the conductivity of the film. The conductivity of the film increases from 0.05 × 10^−7^ S cm^−1^ obtained using H_3_PO_4_ of 75 v% with an irradiation dosage of 5 kGy, and further increases at higher irradiation dosages. For irradiation dosages of 10, 15, and 20 kGy, the values of film conductivity at H_3_PO_4_ of 75 v% are 0.154 × 10^−7^ S cm^−1^, 0.454 × 10^−7^ S cm^−1^, and 0.526 × 10^−7^ S cm^−1^, respectively. Better results are obtained at H_3_PO_4_ 85 v%, at an irradiation dosage of 5 kGy that results in electrical conductivity of 0.21 × 10^−5^ S cm^−1^. The increment of conductivity can be attributed to the doping of H_3_PO_4_ into the PVA thanks to the blending that forms the solution, leading to ionization as such the concentration of H^+^ from H_3_PO_4_ increased in the polymer blend. It implies that at higher H_3_PO_4_ concentrations, the concentration of H^+^ in the polymer blend also increases, which lifts the electrical conductivity.

The insulator nature of the polymer can be shifted as a good conductor after structural modification via doping. The presence of doping in the polymer conjugation system results in exchanges of bonds within the polymer chain. It allows electrons to delocalize within the entire system of polymer [53]. The delocalized electrons move almost freely within the polymer system, acting as carriers of conductive charges [54]. Polymers can be transformed to pose conductivity by allowing the free transfer of electrons between bonds to form anions. Anions and cations react as charge transfer carriers under the influence of the electrical field [55]. Polymer film is typically non-conductive, but the doping process is effective in enhancing its conductivity. Doping allows the flow of electrons to the conductive gap that allows electrons to transfer around the polymer chain. An electrical current is produced when electrons transfer along the polymer chain [43].

The experimental data of polymer film samples demonstrate that the increment in irradiation dosage plays important role in increasing the conductivity. It means that the irradiation mechanism is effective in enhancing the electrical property of polymer, on top of other chemical and physical methods, such as heating. Gamma ray (γ) irradiation can modify the structure of polymers. The interaction between the polymer film and ionization radiation yields changes in physical and chemical properties depending of the dosage.

Table 2 shows three types of PVA-based polymer and their electrical conductivity. It clearly shows the change of the polymer film property from insulator (insulating polymer) into semiconductor σ ≈ 10^−5^ S·cm^−1^ due to gamma ray irradiation, as provided in classification stated elsewhere [56]. It can be stated that the polymer film blend of PVA–H_3_PO_4_ irradiated by gamma ray is a semiconductor material with a conductivity of σ ≈ 10^−5^ S·cm^−1^.

## 4. Conclusions

The polymer film blend of PVA–H_3_PO_4_ irradiated by γ-rays gave a positive response to the optical properties and conductivity by increasing the concentration of H_3_PO_4_ and γ-rays irradiation dose. The values of optical absorption, absorption edge, and energy gap decrease with an increasing H_3_PO_4_ concentration and radiation dose. The irradiation of γ-ray intensifies the interaction between PVA and H_3_PO_4_, leading to an increase in the conductivity of the polymer film.

## Figures and Tables

**Table 1:** Electrical resistivity and conductivity of the H_3_PO_4_ 75 v% polymer film sample.

Doses (kGy)	Resistivity *ρ* (Ω·cm)	Conductivity σ (S cm^−1^)
5	(20.0 ± 0.6) × 10^7^	(0.050 ± 0.6) × 10^−7^
10	(6.5 ± 0.8) × 10^7^	(0.154 ± 0.8) × 10^−7^
15	(2.2 ± 0.4) × 10^7^	(0.454 ± 0.4) × 10^−7^
20	(1.9 ± 0.5) × 10^7^	(0.526 ± 0.5) × 10^−7^

**Table 2:** Conductivity polyvinyl alcohol-based film.

Polymer Film	Electrical Conducitivity
PVA	Dry PVA, σ = 10^−10^–10^−14^ S cm^−1^ PVA in water, σ ≈ 10^−9^ S cm^−1^ [14]
PVA + H_3_PO_4_	Increased in conductivity of six-fold of the pure PVA (σ ≈ 10^−9^ S·cm^−1^) [14]
(PVA + H_3_PO_4_) + Gamma irradiation	H_3_PO_4_ 85 v%, irradiation 5 kGy, σ = (0.21 ± 0,4) × 10^−5^ S cm^−1^ H_3_PO_4_ 75 v%, irradiation 5 kGy, σ = (0.050 ± 0,6) × 10^−7^ S cm^−1^

## Data Availability

The data presented in this study are available on request from the corresponding author.
